# *NtMYB12* Positively Regulates Flavonol Biosynthesis and Enhances Tolerance to Low Pi Stress in *Nicotiana tabacum*

**DOI:** 10.3389/fpls.2019.01683

**Published:** 2020-01-24

**Authors:** Zhaopeng Song, Yong Luo, Weifeng Wang, Ningbo Fan, Daibin Wang, Chao Yang, Hongfang Jia

**Affiliations:** ^1^College of Tobacco Science, Henan Agricultural University, Zhengzhou, China; ^2^Guangxi Branch of China National Tobacco Corporation, Nanning, China; ^3^Key Laboratory of Tobacco Biology & Processing in Ministry of Agriculture, Qingdao, China; ^4^Chongqing Branch of China National Tobacco Corporation, Chongqing, China

**Keywords:** *NtMYB12*, low Pi stress, flavonol biosynthesis, overexpression, *Nicotiana tabacum*

## Abstract

Phosphorus (P) is an essential macronutrient for plant growth and development. The concentration of flavonol, a natural plant antioxidant, is closely related to phosphorus nutritional status. However, the regulatory networks of flavonol biosynthesis under low Pi stress are still unclear. In this study, we identified a PFG-type MYB gene, *NtMYB12*, whose expression was significantly up-regulated under low Pi conditions. Overexpression of *NtMYB12* dramatically increased flavonol concentration and the expression of certain flavonol biosynthetic genes (*NtCHS*, *NtCHI*, and *NtFLS*) in transgenic tobacco. Moreover, overexpression of *NtMYB12* also increased the total P concentration and enhanced tobacco tolerance of low Pi stress by increasing the expression of *Pht1*-family genes (*NtPT1* and *NtPT2*). We further demonstrated that *NtCHS-*overexpressing plants and *NtPT2-*overexpressing plants also had increased flavonol and P accumulation and higher tolerance to low Pi stress, showing a similar phenotype to *NtMYB12*-overexpressing transgenic tobacco under low Pi stress. These results suggested that tobacco *NtMYB12* acts as a phosphorus starvation response enhancement factor and regulates *NtCHS* and *NtPT2* expression, which results in increased flavonol and P accumulation and enhances tolerance to low Pi stress.

## Introduction

Flavonoids are a large group of secondary metabolites in plants, generally having a C6-C3-C6 carbon skeleton (Halbwirth, 2010; Li et al., 2015). The most common flavonoids found in plants are anthocyanin and flavonols (Holton and Edwina, 1995; Jia et al., 2015). Although various flavonol biofunctions in plants have been recognized, antioxidant capacity is a common feature for flavonols and most other flavonoid compounds (Nijveldt et al., 2001; Kuhn et al., 2011). Previous reports have shown that flavonols play important roles in various biological processes in plants, such as resistance to UV-B damage, cell-wall formation, and defense against pathogens, implying that flavonols play a key role in plant biofunction under stress conditions (Winkel, 2001; Khan et al., 2010; Xu et al., 2014; Martinez et al., 2016).

Flavonoid biosynthesis has been well documented in *Arabidopsis*, tomato, and woody plants (Holton and Edwina, 1995; Niggeweg et al., 2004; Li et al., 2015; Zhai et al., 2016; Zhai et al., 2019). Chalcone synthase (*CHS*), chalcone isomerase (*CHI*), flavanone 3-hydroxylase (*F3H*), flavonol synthase (*FLS*), and dihydroflavonol 4-reductase (*DFR*) catalyze the biosynthetic steps that convert phenylpropanoid precursors to flavonols and anthocyanin. Some MYB transcriptional factors (TFs) have been shown to directly regulate the biosynthesis of flavonoids (Dubos et al., 2010; Wang et al., 2013; Li, 2014; Liu et al., 2015; Zhai et al., 2016; Zhai et al., 2019). *MYB75, MYB90, MYB113*, and *MYB114* are products of anthocyanin pigment (PAP)-type MYBs and are reported to regulate anthocyanin biosynthesis in *Arabidopsis* (Borevitz et al., 2000; Nesi et al., 2001; Gonzalez et al., 2008)*. MYB11, MYB12, MYB111*, and *HY5* are reported to regulate flavonol biosynthesis in *Arabidopsis* (Mehrtens et al., 2005; Stracke et al., 2010). Recently, Zhai et al. found that two MYB genes, *PbMYB9* and *PbMYB12*, which are among the products of the flavonol glycoside (PFG)-type MYB TF family, are positive regulators of the flavonol biosynthesis pathway in pears (*Pyrus bretschneideri Rehd*.) and work by activating the expression of genes encoding *CHS* and *FLS* (Zhai et al., 2016; Zhai et al., 2019). Although the functions and regulatory mechanisms of plant MYB genes have been widely studied, more research is needed to understand the biological role of each member of this vast TF family.

Inorganic phosphorus (Pi) is an essential macronutrient for plant growth and development. It has not only a structural role in DNA, RNA, ATP, and phospholipids, but also an important regulatory role in several physiological processes, including photosynthesis, respiration, signal transduction, and energy metabolism (Marschner, 1995; Vance et al., 2003). P is typically absorbed by Pi transporters (PTs) and assimilated into plant cells and tissues in its inorganic form (Pi) (Marschner, 1995). Genes codifying these Pi transporters are generally classified into four families (*Pht1* to *Pht4*), which suggests their diverse biological functions for plant growth and development (Jia et al., 2011; Song et al., 2017; Chang et al., 2019). The *Pht1* family belongs to the high-affinity PTs, whose expression is up-regulated distinctly under low Pi stress conditions, playing a crucial role in both Pi uptake and translocation under Pi deficiency.

Pi deficiency impairs the biosynthesis of macromolecules and various other biological processes, which may result in serious cellular damage and growth retardation (Chang et al., 2019). Plants respond to environmental Pi deficiency by altering their developmental and metabolic programs to better survive and grow under nutritional stress (Franco-Zorrilla et al., 2004; Chiou and Lin, 2011; Lynch, 2011; Smith et al., 2011; Jain et al., 2012; Jia et al., 2017; Pan et al., 2019). Typically, numerous genes are activated (e.g., high-affinity Pi transporters), plant root architecture is changed, anthocyanin is accumulated, and antioxidant capacity is enhanced. Previously, we found that Pi deficiency prompts tobacco plants to accumulate flavonols, implying that there is an interaction between phosphorus nutrition and flavonol metabolism (Jia et al., 2015). However, the molecular process underlying this response is still unclear.

Tobacco is a major cash crop worldwide and has abundant flavonoids in its leaves. For this reason, tobacco is the proper model plant in which to explore the function of flavonoid biosynthetic genes in low Pi tolerance. In our previous study, the transcriptome databases were analyzed for low N, low Pi, and low K stress conditions in tobacco. The results showed that *NtMYB12* expression was induced in the transcriptome database under the Pi-deficient condition, implying that *NtMYB12* may play a key role in the Pi signaling pathway. In this study, we comprehensively investigated the effects of the tobacco gene *NtMYB12* on flavonol biosynthesis and plant growth and development under low Pi stress. Our results showed that *NtMYB12* is a PFG-type MYB transcription factor that may be involved in the Pi signaling pathway. Overexpression of this gene enhances tolerance of low Pi stress by increasing flavonol and P accumulation in tobacco. These data demonstrate that *NtMYB12* plays a critical role in the flavonol-synthesis pathway under low Pi stress conditions.

## Material and Methods

### Plant Material and Growth Conditions

*NtMYB12*-overexpression (T3 generation), *NtCHS*-overexpression (T2 generation), and *NtPT2*-overexpression (T3 generation) transgenic (named *35S:NtMYB12, 35S:NtCHS and 35S:NtPT2*) and wild-type (*Nicotiana tabacum cv*, *Yunyan 87*) tobacco plants were used in this study. Samples of 150 tobacco seeds of transgenic plants and WT were sterilized with a solution of 75% (v/v) ethanol for 30 s and 10% (v/v) sodium hypochlorite for 10 min then washed six times with sterile distilled water and transferred to seedling trays (3 days). The trays were kept in a culture room in a dark environment of 28℃ for proper germination. The germinated seedlings were placed in a light incubator (RXZ-600, Ningbo Jiangnan Instrument Factory, China) with a day/night temperature of 28℃/23℃ and a 14-h-light/10-h-dark photoperiod. The relative humidity was approximately 60%, and the light intensity was controlled to 300 μmol·m^-2^·s^-1^ for 7 days. Sixteen tobacco seedlings were grown in each culture vessel (depth: 5 cm; diameter: 15 cm) with sand, and the Hoagland's nutrient solution was changed every day in the growth chamber (described above). Quarter-strength Hoagland's nutrient solution was used to culture the seedlings in the first 3 days, and half-strength nutrient solution was used in the second 3 days. Full-strength Hoagland's solution with high Pi (HP; 1mM Pi) or low Pi (LP; 0.02mM Pi) was then supplied to culture the seedlings for 21 days. Afterward, the tobacco seedlings were harvested for further analysis. (1) The phenotype of the tobacco seedlings was observed and recorded. (2) Some of the leaves were used for NBT staining. (3) The enzyme activity and MDA concentration of the seedlings were measured. (4) Some tobacco seedlings were used to detect the flavonol concentration, Pi concentration, and gene expression. (5) The other tobacco seedlings were oven-dried and used to measure the total P concentration.

### Isolation and Sequence Analysis of *NtMYB12*

The full-length entire coding sequences (CDS) of *NtMYB12* (GenBank accession: XM_016624824) and promoter from tobacco “Yunyan 87” were cloned using specific primers. The primers were designed based on the published sequence data in the tobacco Genome Database (**Table S1**). Cis elements in the promoters were identified *via* PlantCare (http://bioinformatics.psb.ugent.be/webtools/plantcare/html/). Sequences of multiple peptides were aligned with DNAMAN software (Lynnon Biosoft, United States). A phylogenetic tree was constructed using the Neighbor-Joining (NJ) algorithm in MEGA7.0 software (Kumar et al., 2008).

### Sub-Cellular Localization of *NtMYB12*

For the analysis of subcellular localization, the entire coding sequence (CDS) of *NtMYB12* was amplified and used to construct an N-terminus green fluorescent protein (GFP) fusion vector, pMDC43-NtMYB12. The construct was transiently introduced into tobacco epidermal cells by injection, and the same transformation was carried out with *P35S::GFP* for control cells. After transformation, the tobacco epidermis samples were kept in a dark environment (25℃/16h). GFP fluorescence was observed using a confocal laser scanning microscope (Nikon C2-ER).

### Generation of Transgenic Plants

For the preparation of cassettes for the overexpression of *NtMYB12*, *NtCHS*, and *NtPT2*, each gene was amplified and synthesized from tobacco leaf cDNA as follows. To construct the overexpression vector NtMYB12- pCAMBIA1305, the entire coding sequence (CDS) of *NtMYB12* was amplified using primers (**Table S1**). The PCR product was digested with SacI/KpnI and cloned into the SacI/KpnI-digested pCAMBIA1305 plasmid under the control of the CaMV 35S promoter. In order to construct the overexpression vector NtCHS-pCAMBIA1305, the coding region (CDS) of *NtCHS* (Chen et al., 2017) was amplified using primers (**Table S1**). The PCR product was digested with SacI/KpnI and cloned into the SacI/KpnI-digested pCAMBIA1305 plasmid under the control of the CaMV 35S promoter. To construct the overexpression vector NtPT2-pCAMBIA1305, the coding region (CDS) of *NtPT2* (Jia et al., 2018) was amplified using primers (**Table S1**). The PCR product was digested with SacI/XhoI and cloned into the SacI/XhoI-digested NtPT2-pCAMBIA1305 plasmid under the control of the CaMV 35S promoter. All of the constructs were transferred to *Agrobacterium tumefaciens* strain EHA105 by electroporation and then transformed into tobacco (*Nicotiana tabacum cv, Yunyan 87*) as described previously (Chen et al., 2011; Jia et al., 2018).

### Southern Blotting Analysis

The independent *35S:NtMYB12* transgenic tobacco lines were identified by Southern blotting analysis. Genomic DNA was extracted from the leaves of wild-type (WT) and T1 transgenic plants by the SDS method, and 5 μg was digested with the restriction enzyme SacI overnight at 37°C. The digested DNA was separated on a 0.8% (w/v) agarose gel and then transferred to a Hybond-N^+^ nylon membrane and hybridized with the coding sequence of the hygromycin-resistance gene, which was used as the hybridization probe, as described previously (Zhou et al., 2008; Jia et al.,2017).

### Determination of Flavonoid Compounds

The major flavonols were extracted from the leaves of tobacco samples (0.2 mg) using 2 ml of 100% methanol from Sigma (http://www.sigmaaldrich.com/) (fresh-weight basis). The flavonols were detected by HPLC (high-performance liquid chromatography, Agilent Technologies 1200 series) with a column (Agilent Technologies ZORBAX SB-C18 4.6 mm × 250 mm) and quantified by comparing the area of each individual peak with the curves obtained for the individual compounds (Li et al.,2015).

This was equilibrated with solvent A (water/acetonitrile/formic acid, 87:3:10) and solvent B (water/acetonitrile/formic acid, 40:50:10), eluting with a gradient of increasing solvent B at a flow rate 1ml·min^-1^. The gradient conditions were: time 0, 96% A, 4% B; 20 min, 80% A, 20% B; 35 min, 60% A, 40% B; 40 min, 40% A, 60% B; 45 min, 10% A, 90% B; 55 min, 96% A, 4% B. Detection by ultraviolet (UV) chromatograms was recorded at 325 nm. All of the flavonol standards, rutin, and kaempferolrutinoside were obtained from either Sigma-Aldrich or Extrasynthèse (Genay, France).

### Determination of Total Anthocyanin Concentration

Total anthocyanin was extracted from fresh leaves of tobacco plants in 1% HCl in methanol with gentle shaking in the dark at 4℃ overnight. The extracts were diluted with an equal volume of water. An equal volume of chloroform was then added to the extracts, and the samples were vortexed slowly for a few seconds. The supernatant aqueous/methanol phase was obtained by centrifugation at 12,000g for 10 min. The absorbance of the supernatant at 530 nm was recorded in order to calculate the concentration of anthocyanin (Bariola et al., 1999; Li et al., 2014). Five repeats were conducted for each of three biological replicates. The values were normalized to the fresh weight of each sample.

### RNA Extraction, cDNA Synthesis, and RT-qPCR

Total RNA extraction and cDNA synthesis were carried out using the method described by Jia et al. (2018). Reverse transcription polymerase chain reaction (RT-qPCR) analysis was performed using gene-specific primers for flavonol synthesis-related genes and *Pht1* family genes in tobacco. The primers used for qRT-PCR are listed in **Table S1**. Relative transcript levels were normalized to that of *NtL25* (L18908.1) and presented as 2^-ΔΔCt^ (Livak and Schmittgen, 2001).

### Biochemical Staining and Physiological Measurements

Superoxide (O_2_-) staining was performed by infiltration by the nitro blue tetrazolium (NBT) method (Liu et al., 2018; Song et al., 2019). SOD and CAT activities were measured as described previously (Wang et al., 2012). The MDA concentration was determined with the thiobarbituric acid (TBA) method (Luo et al., 2019).

### Measurement of Total P and Soluble Pi Concentration in Tobacco

About 0.05 g of dry ground powder of each sample was used for the measurement of total P concentration in the plants by the method described previously (Jia et al., 2015; Jia et al., 2018). About 0.5 g of fresh samples were used for the measurement of soluble Pi concentration in the plants by the method described previously (Zhou et al., 2008).

### Statistical Analysis

All data were statistically analyzed by two-way ANOVA analysis and Tukey's multi-comparisons test (P < 0.05) in SAS software version 8.1. The results were expressed as the means and the corresponding standard errors.

## Results

### Identification and Molecular Characterization of *NtMYB12*

**Figure1:**
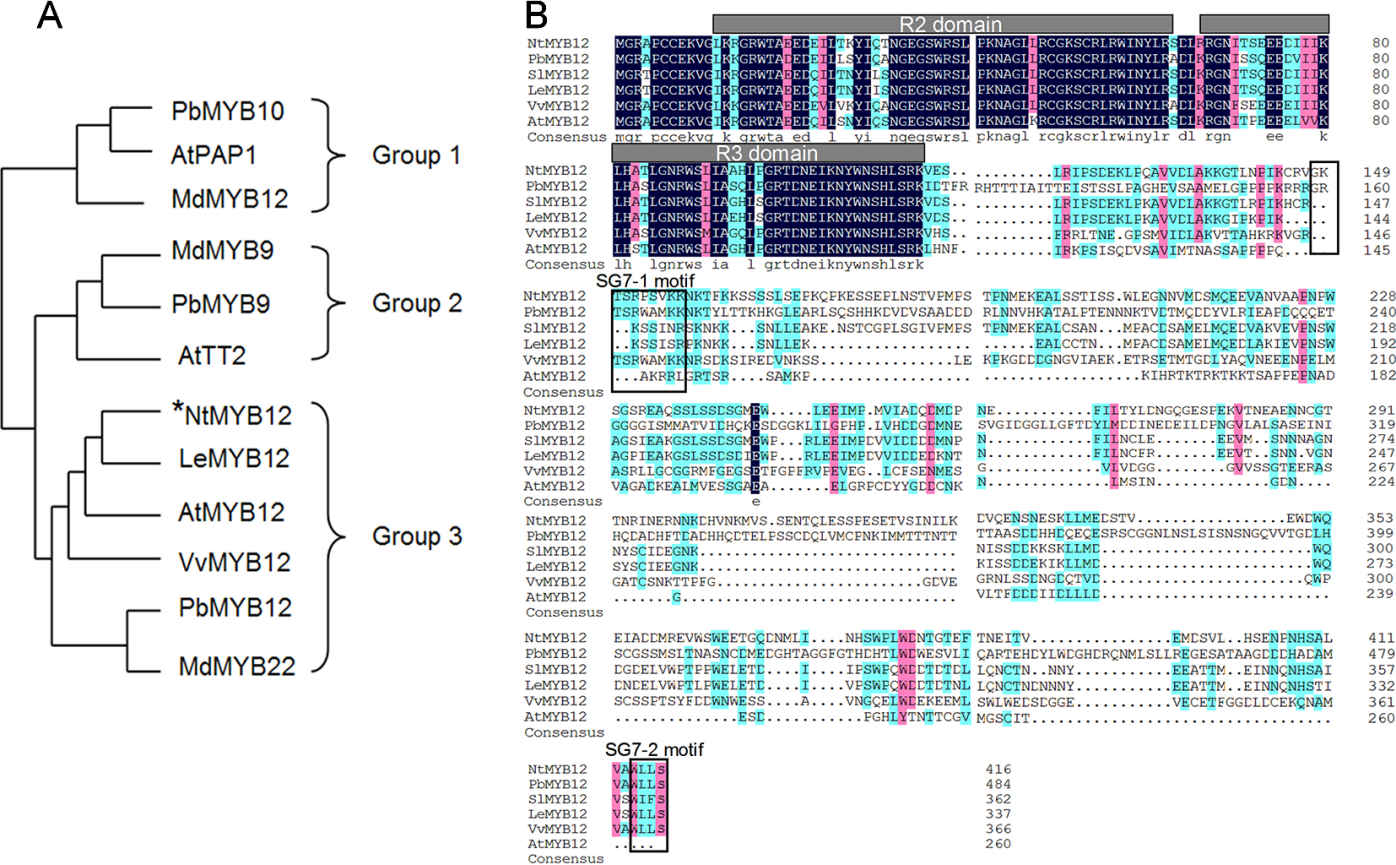
Sequence alignment and phylogenetic analysis of *NtMYB12*. **(A)** Phylogenetic analysis of typical flavonoid-regulating MYBs from different species. These MYBs were classified into three major groups. The MYB protein sequences in other species were obtained from the NCBI database. Their protein accessions in the NCBI database are as follows: pear *PbMYB10* (ALU57825.1), Arabidopsis *PAP1* (AEE33419.1), apple *MdMYB12* (ADL36755.1), apple *MdMYB9* (ABB84757.1), pear *PbMYB9* (ALU57827.1), Arabidopsis *TT2* (OAO91653.1), Arabidopsis *MYB12* (ABB03913), grapevine *VvMYB12* (XP_002269995), *PbMYB12* (ALF95174.1), and apple *MdMYB22* (AAZ20438.1). The phylogenetic tree was constructed by MEGA7.0 using a bootstrap test of phylogeny with the Neighbor-Joining test and default parameters. **(B)** Protein sequence alignments of PFG-type MYBs from different species. The conserved R2 and R3 domains are underlined. Previously described SG7–1 and SG7–2 motifs are boxed. The multiple alignments were performed using DNAMAN 6.0.

To determine the function of *NtMYB12*, the full-length coding sequence (CDS) of *NtMYB12* was cloned from tobacco. The length of the protein encoded by *NtMYB12* was 417 amino acids. Evolutionary study of the MYB gene family indicates that typical MYB transcription factors (TFs) involved in flavonoid biosynthesis can be classified into three different groups: PAP-type MYB TFs (group I, anthocyanin regulators), TT2-type MYB TFs (group II, flavanol regulators), and PFG-type MYB TFs (group III, flavonol regulators). Notably, *NtMYB12* clusters with the PFG-type MYB TFs in group III (**Figure 1A**). Protein sequence alignment analysis showed that *NtMYB12* contains two characteristic motifs, SG7-1 and SG7-2, commonly found in PFG-type MYB TFs (**Figure 1B**). Thus, *NtMYB12* was identified as a typical PFG-type MYB TF, suggesting that *NtMYB12* may be involved in the regulation of flavonol biosynthesis in tobacco.

### Subcellular Localization of *NtMYB12*

**Figure 2:**
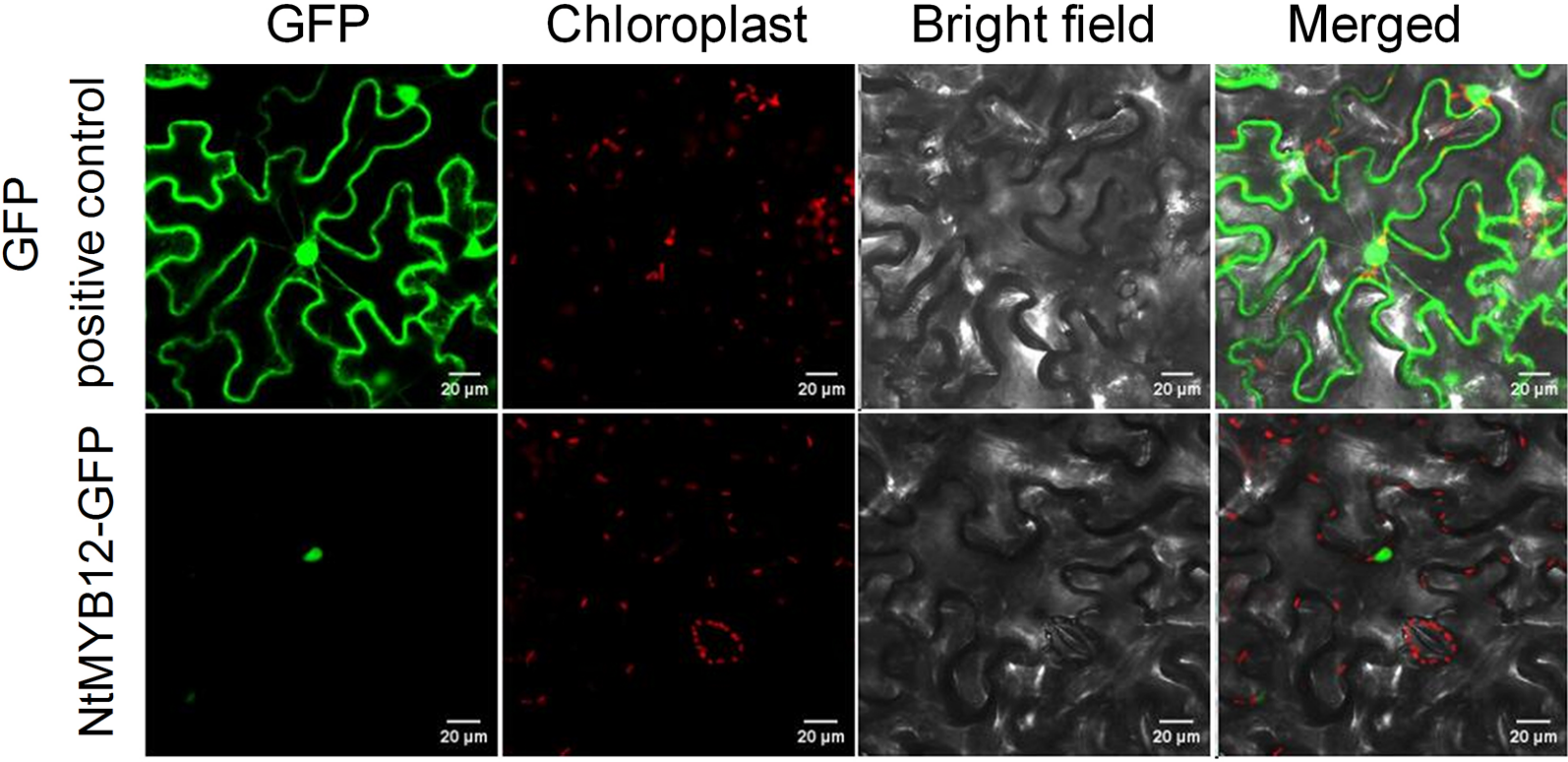
Sub-cellular localization of *NtMYB12* in tobacco epidermal cells. The fusion protein (*NtMYB12*-GFP) and GFP-positive control were independently transiently expressed in tobacco cells. GFP fluorescence was observed with a fluorescence microscope.

Plant MYB family members were predicted to be localized in the nucleoplasm. To verify *NtMYB12* subcellular localization, we constructed N-terminal GFP fusions driven by the cauliflower mosaic virus 35S promoter and transfected the derived expression vector into tobacco epidermal cells. As expected, microscopic observations demonstrated that the fused protein (NtMYB12-GFP) was restricted to the nucleoplasm, whereas, in control transgenic cells expressing *p35S::GFP*, the GFP signal was detected throughout all the cell (**Figure 2**), suggesting that *NtMYB12* may work as a transcription factor.

### Expression of *NtMYB12* was Up-Regulated by Low Pi Treatment

**Figure 3:**
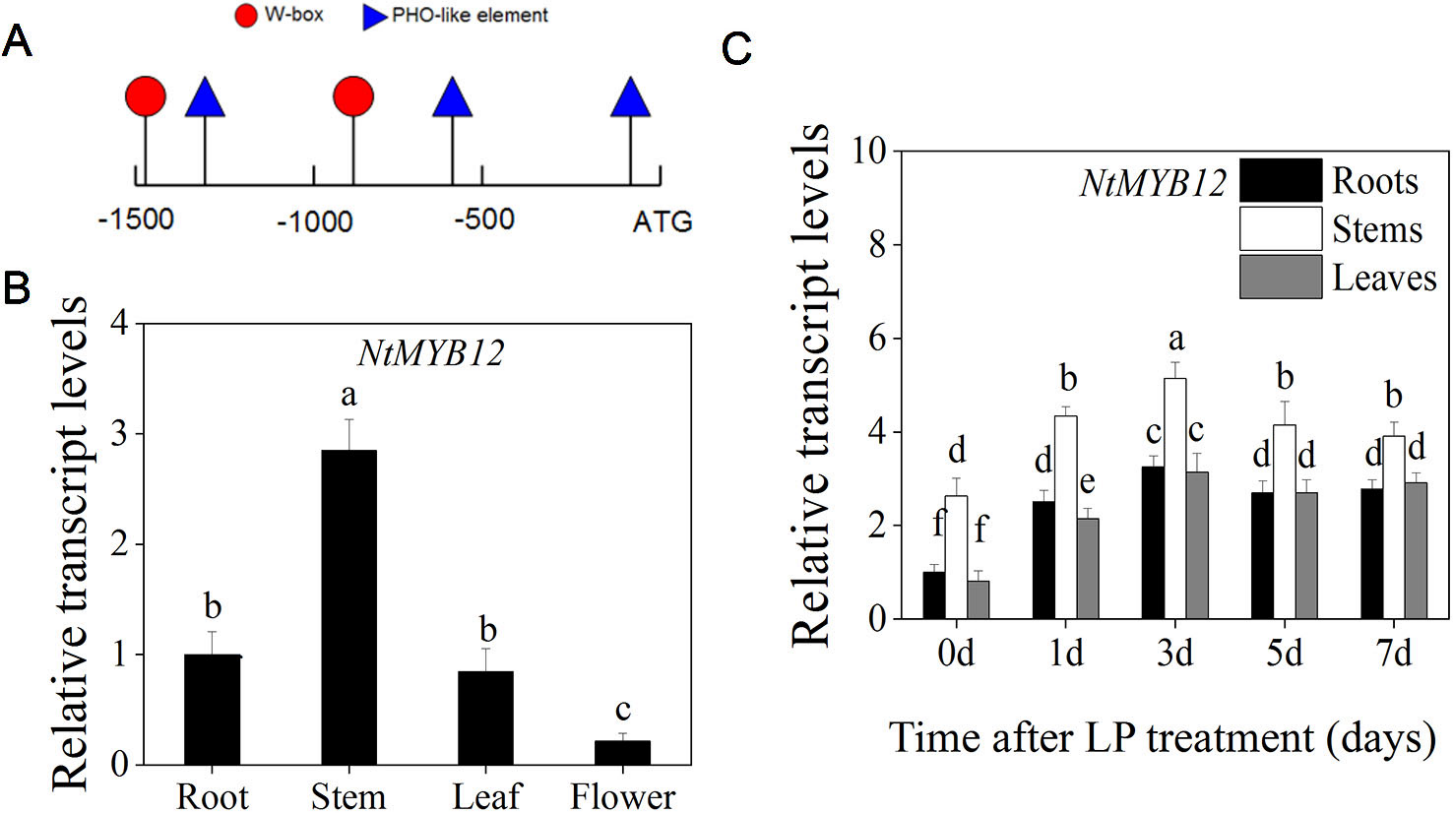
Expression patterns of *NtMYB12* in different organs and effects of Pi deprivation on the expression of *NtMYB12* in tobacco. **(A)** Analysis of the *NtMYB12* promoter. **(B)** Relative transcript levels of *NtMYB12* in different tissues of tobacco. **(C)** Time course of *NtMYB12* expression in response to low Pi stress. Thirty-day old seedlings were grown in nutrient solution with low Pi (LP; 0.02mM Pi) for 7 days. Data are the means ± SDs of five biological replicates. Different letters indicate significant differences (*P* < 0.05).

To determine whether *NtMYB12* is regulated by Pi deficiency, the promoter sequence of *NtMYB12* in tobacco was analyzed using PlantCare (Lescot et al., 2002). The low Pi-responsive elements W-box and PHO-like were found in the *NtMYB12* promoter (**Figure 3A**), indicating that *NtMYB12* might be involved in the Pi signaling pathway in tobacco.

We further checked the expression of *NtMYB12* in the roots, stems, leaves, and flowers of tobacco. *NtMYB12* was expressed in all of the organs examined, with the highest levels in stems and the lowest levels in flowers (**Figure 3B**). Moreover, the expression of *NtMYB12* was significantly increased under low Pi conditions in roots, stems, and leaves up to 7 days (**Figure 3C**), implying that this gene may play a regulatory role in tobacco response and adaptation to low Pi levels.

### Overexpression of *NtMYB12* Enhanced Tobacco Tolerance to Low Pi Stress

**Figure 4:**
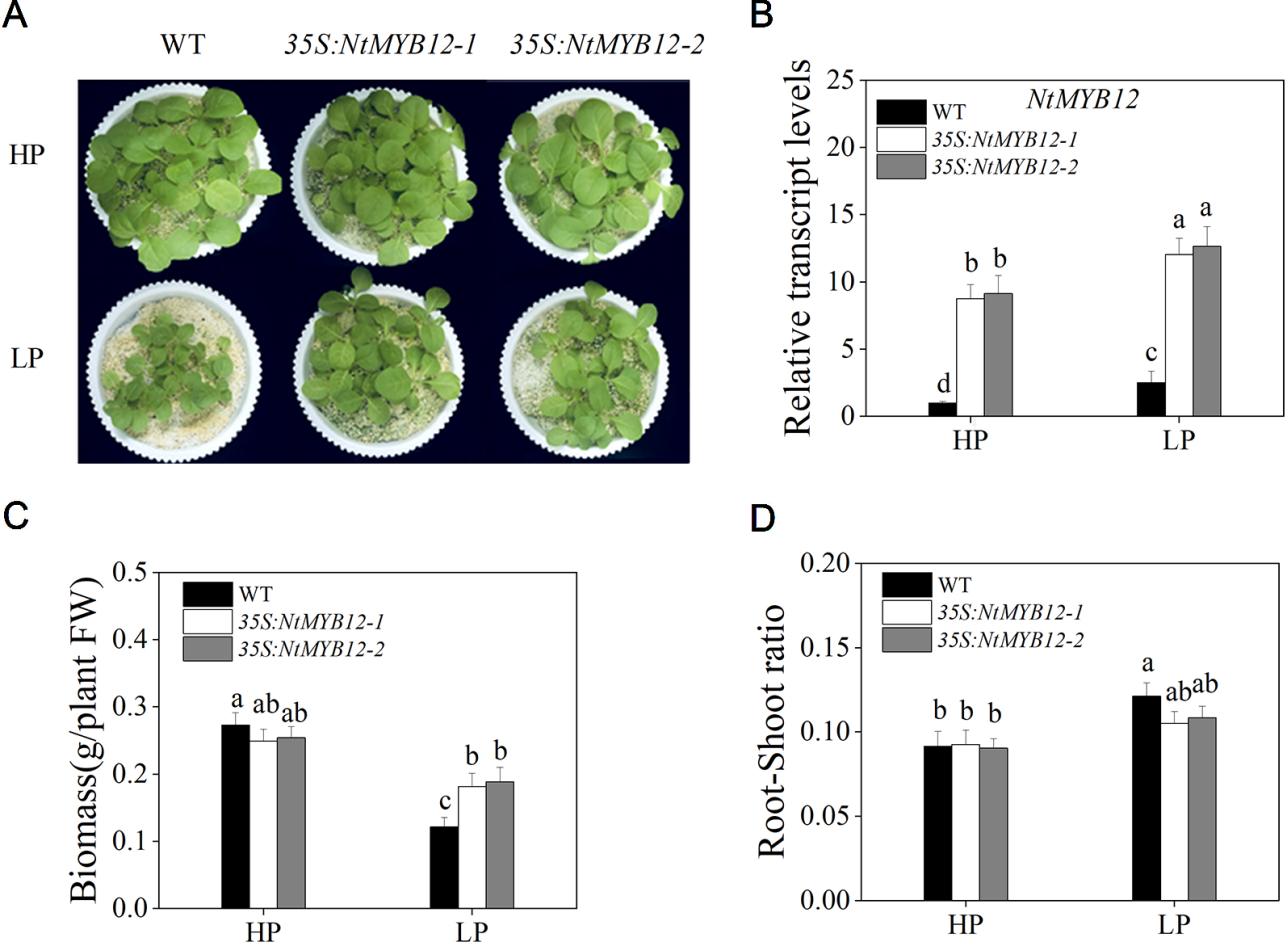
Expression of *NtMYB12* in transgenic tobacco and characterization of the WT and transgenic tobacco. **(A)** Characterization of the WT and transgenic tobacco under HP and LP conditions. **(B)** Relative transcript levels of *NtMYB12* in WT and transgenic plants. **(C)** Biomass of the WT and transgenic plants under HP and LP conditions. **(D)** Root–shoot ratio of the WT and transgenic plants under HP and LP conditions. Sixteen-day-old seedlings were transferred to the full-strength culture solution and supplied with high Pi (HP; 1mM Pi) or low Pi (LP; 0.02mM Pi) for 21 days. FW, fresh weight. Data are the means ± SDs of five biological replicates. Different letters indicate significant differences (*P* < 0.05).

To determine the function of *NtMYB12*, twenty transgenic tobacco lines were generated by introducing the *NtMYB12*-overexpression construct. Two independent transgenic lines (named *35S:NtMYB12-1* and *35S:NtMYB12-2*) were identified by Southern blot analysis and used in this study (**Figure S1** and **Figure 4**). Under Pi-sufficient conditions (HP; 1 mM Pi), the phenotype and biomass of *35S:NtMYB12* plants were not significantly different from those of wild-type (WT) plants (**Figure 4A**). Under Pi-deficient conditions (LP; 0.02 mM Pi), the biomass in *35S:NtMYB12* plants was significantly higher than in WT plants (**Figures 4B**, **C**). However, we also found that the root–shoot ratios were not significantly different between *35S:NtMYB12* plants and WT under low Pi stress (**Figure 4D**).

**Figure 5:**
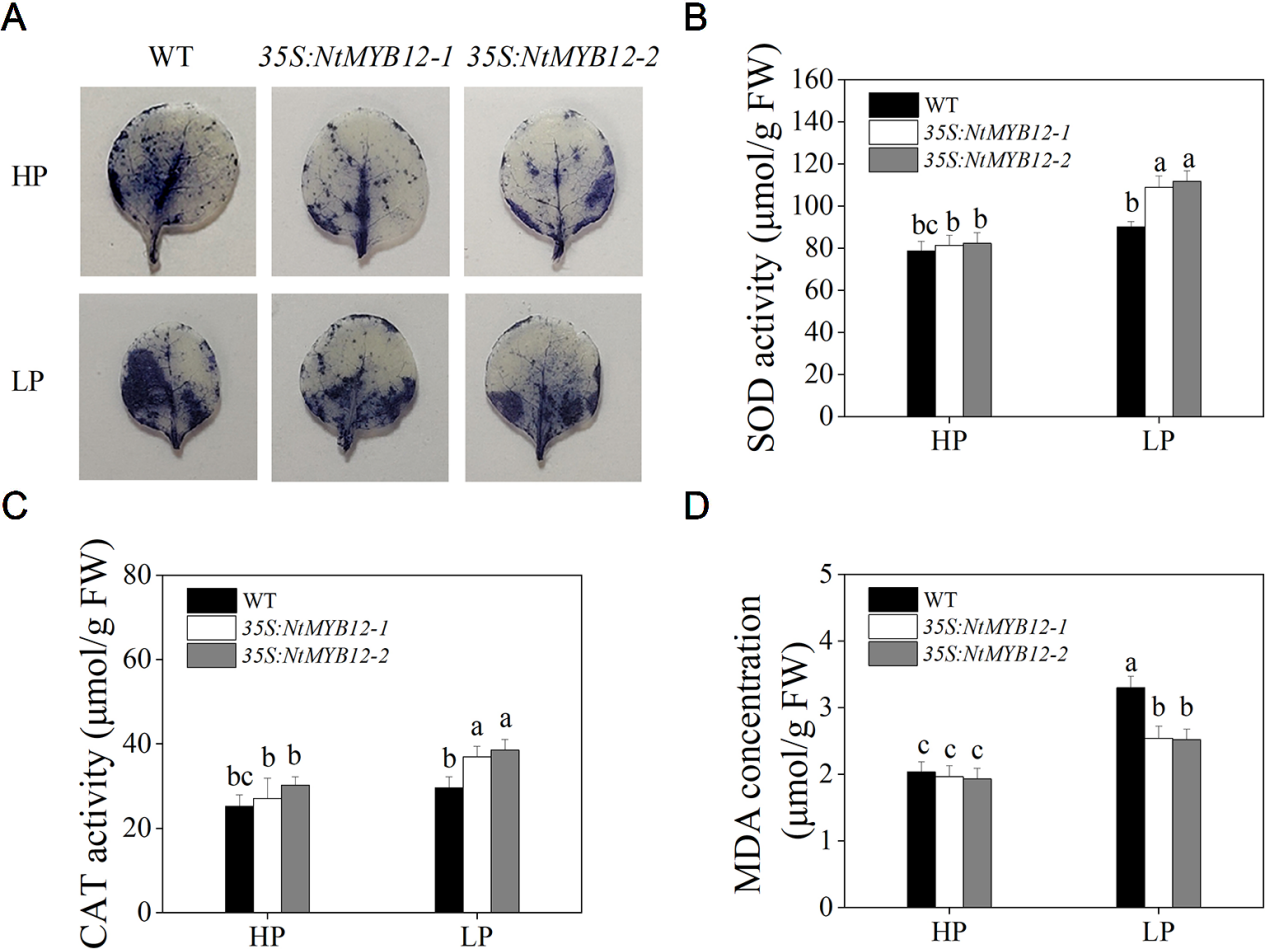
Effects of P treatment on antioxidant enzymes of WT and transgenic tobacco leaves. **(A)** Detection of Superoxide (O_2_-) by NBT staining in tobacco seedlings. **(B)** SOD activity of the WT and transgenic plants. **(C)** CAT activity of the WT and transgenic plants. **(D)** MDA concentration of the WT and transgenic plants. Sixteen-day-old seedlings were transferred to the full-strength culture solution and supplied with high Pi (HP; 1mM Pi) or low Pi (LP; 0.02mM Pi) for 21 days. FW, fresh weight. Data are the means ± SDs of five biological replicates. Different letters indicate significant differences (*P* < 0.05).

**Figure 6:**
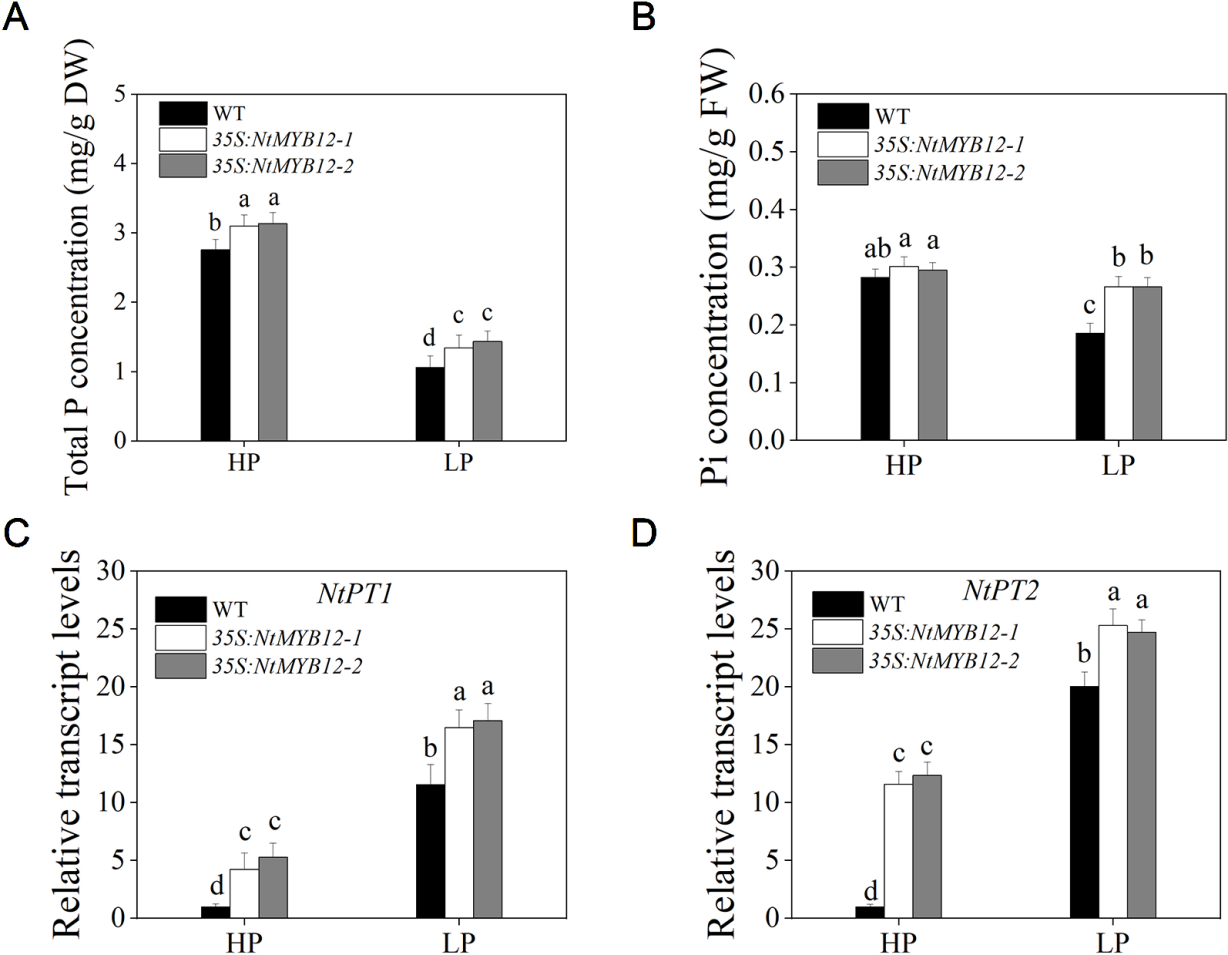
Total P, Pi concentration, and relative transcript levels of two members of the tobacco *Pht1* family in WT and *35S:NtMYB12* plants under HP and LP conditions. **(A**–**B)** Total P and Pi concentration of WT and transgenic plants in tobacco. **(C**–**D)** Effect of *NtMYB12* overexpression on relative transcript levels of *NtPT1* and *NtPT2* in tobacco under HP and LP conditions. Sixteen-day-old seedlings were transferred to the full-strength culture solution and supplied with high Pi (HP; 1mM Pi) or low Pi (LP; 0.02mM Pi) for 21 days. The whole plants of the WT and transgenic plants were used for measuring Total P and Pi concentration and for RNA extraction. FW, fresh weight; DW, dry weight. Data are the means ± SDs of five biological replicates. Different letters indicate significant differences (*P* < 0.05).

To determine the antioxidant capacity of *35S:NtMYB12* plants under HP and LP conditions, we investigated the accumulation of superoxide (O_2_-) by nitroblue tetrazolium (NBT) staining. A large amount of this ROS was detected in the leaves of tobacco WT plants but not in transgenic plants (**Figure 5A**). We also checked leaf concentrations of SOD, CAT, and MDA and found that *NtMYB12* overexpression in tobacco significantly increased the plant antioxidant capacity (**Figures 5B–D**). Total P concentration and *Pht1* family gene expression in *35S:NtMYB12* plants under HP and LP conditions were also assessed. Plants overexpressing *NtMYB12* had higher P concentrations under both HP and LP conditions (**Figure 6**); this finding was consistent with the expression pattern of genes belonging to the *Pht1* family. All of these results suggested that *NtMYB12* might play a crucial role under low Pi stress conditions.

### Overexpression of *NtMYB12* Resulted in Flavonol Accumulation in Both Pi-Deficient and Pi-Sufficient Conditions

**Figure 7:**
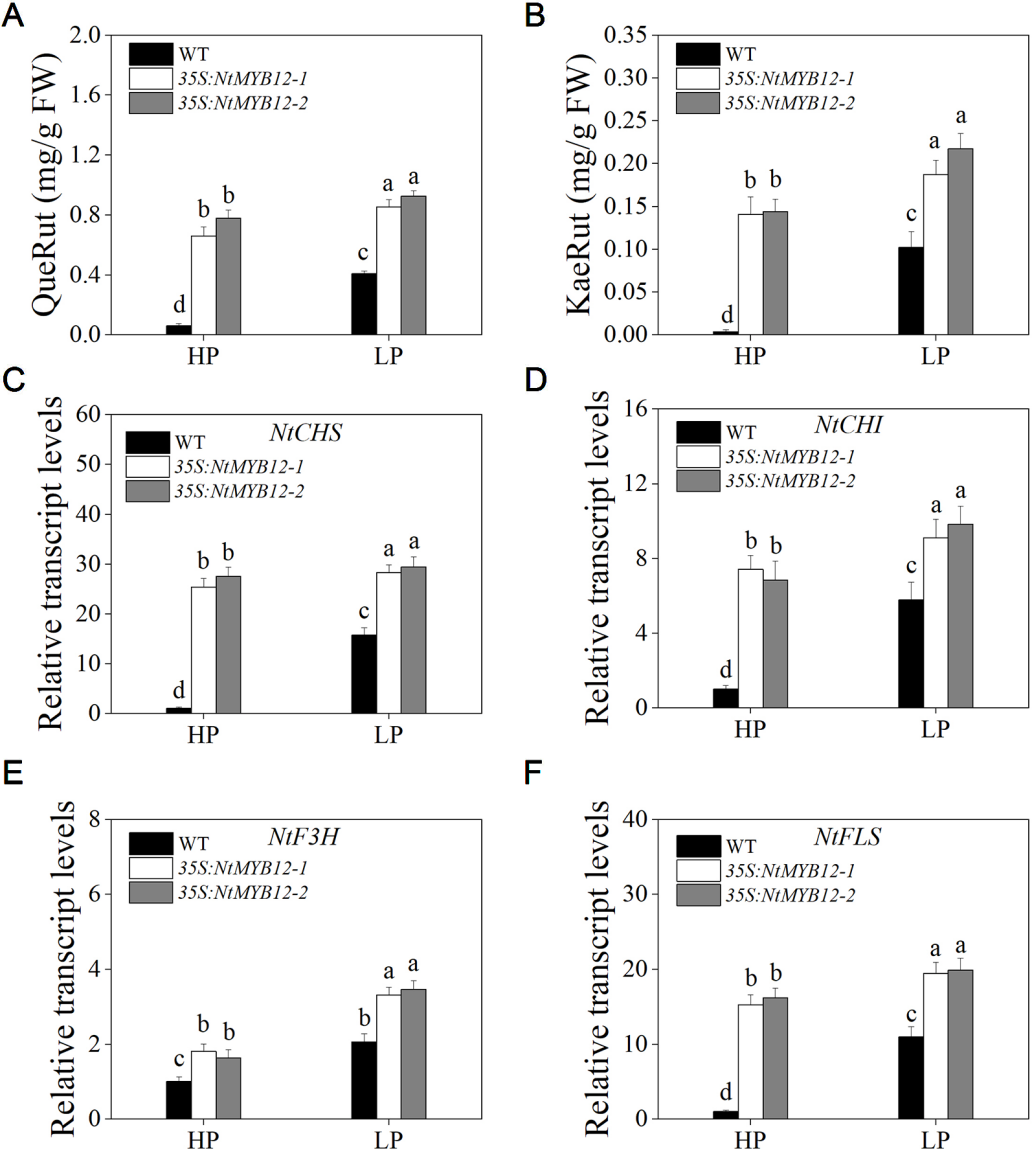
Quantification of flavonol concentration and relative transcript levels of majority genes encoding flavonoid biosynthetic enzymes in WT and *35S:NtMYB12* plants under HP and LP conditions. **(A**–**B)** QueRut and KaeRut concentrations of WT and overexpression tobacco plants. **(C**–**F)** Effect of *NtMYB12* overexpression on relative transcript levels of flavonol biosynthetic genes in tobacco. Sixteen-day-old seedlings were transferred to the full-strength culture solution and supplied with high Pi (HP; 1mM Pi) or low Pi (LP; 0.02mM Pi) for 21 days. The whole plants of the WT and transgenic plants were used for RNA extraction. FW, fresh weight; *CHS*, chalcone synthase; *CHI*, chalcone isomerase; *F3H*, flavanone 3-hydroxylase; *FLS*, flavonol synthase. Data are the means ± SDs of five biological replicates. Different letters indicate significant differences (*P* < 0.05).

The function of flavonol biosynthesis in tobacco was investigated by measuring the flavonol concentration in the leaves of *35S:NtMYB12* and WT tobacco plants growing under Pi-deficient and Pi-sufficient conditions. The major flavonols (rutin and kaempferol rutinoside) were detected in both the wild-type and overexpressing plants. In *35S:NtMYB12* leaves, flavonol concentration was 10-fold higher than in WT under HP conditions (**Figures 7A**, **B**). Under LP conditions, the *35S:NtMYB12* plants showed a significantly increased flavonol concentration while WT plants did not, implying that *NtMYB12* is involved in the flavonol biosynthetic pathway and Pi signaling pathway in tobacco.

To detect whether *NtMYB12* is involved in Pi-supply-regulated flavonol biosynthesis, we analyzed the expression levels of genes involved in flavonol biosynthesis. We found that the expression levels of the genes *CHS*, *CHI*, and *FLS* in the leaves of *35S:NtMYB12* plants were significantly higher than in the WT plants under HP conditions, suggesting increased flavonol biosynthesis in the *35S:NtMYB12* plants compared with that in the WT plants (**Figures 7C**–**F**).

### *NtCHS*- and *NtPT2*-Overexpressing Transgenic Plants Enhanced Low Pi Tolerance

**Figure 8:**
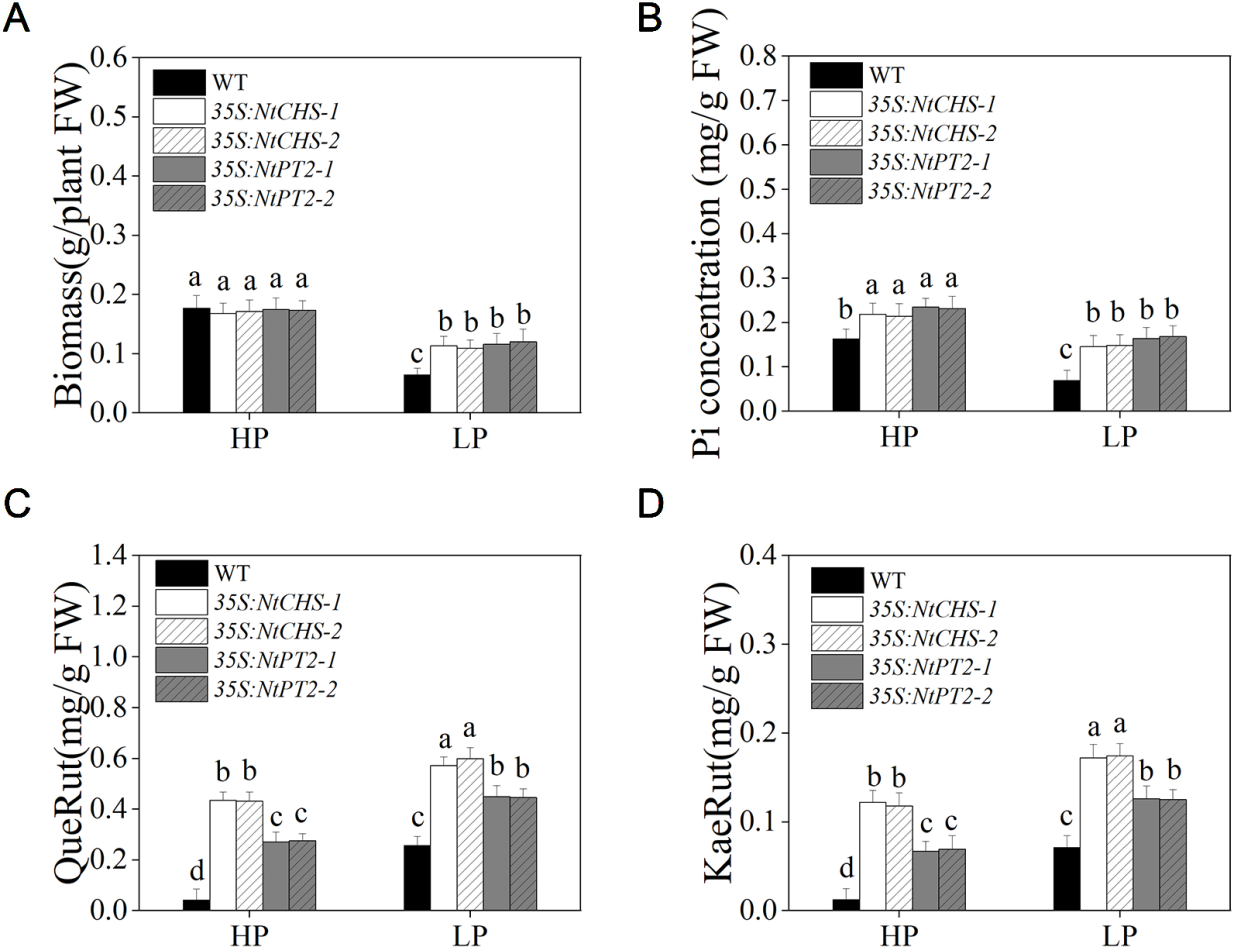
Biomass, Pi concentration, and flavonol concentration in WT, *35S:NtCHS*, and *35S:NtPT2* transgenic plants under HP and LP conditions. **(A**–**B)** Biomass and Pi concentration of the WT and transgenic plants under HP and LP conditions. **(C**–**D)** QueRut and KaeRut concentrations of the WT and transgenic tobacco plants. Sixteen-day-old seedlings were transferred to the full-strength culture solution and supplied with high Pi (HP; 1mM Pi) or low Pi (LP; 0.02mM Pi) for 14 days. FW, fresh weight. Data are the means ± SDs of three biological replicates. Different letters indicate significant differences (*P* < 0.05).

To further determine whether *NtMYB12* plays a critical role in regulating the transcription of flavonol biosynthesis-related genes and *Pht1* family genes, *NtCHS*-overexpressing independent transgenic lines (named *35S:NtCHS*-*1* and *35S:NtCHS*-*2*) and *NtPT2*-overexpressing independent transgenic lines (named *35S:NtPT2*-*1* and *35S:NtPT2*-*2*) were selected for further analyses. The expression levels of *NtCHS* and *NtPT2* were significantly up-regulated under low Pi stress conditions in WT plants, implying that the two genes play a key role in the Pi-starvation signaling pathway (**Figure S2**). We further detected the biomass, Pi concentration and flavonol concentration of *35S:NtCHS* and *35S:NtPT2* plants under HP and LP conditions. Interesting, the results showed that the *35S:NtCHS* and *35S:NtPT2* transgenic tobacco also had a similar phenotype to *NtMYB12* transgenic tobacco, such as increased flavonol and Pi concentrations and higher tolerance to low Pi stress under low Pi conditions (**Figure 8** and **Figure S2**), suggesting that *NtCHS* and *NtPT2* were the key downstream target genes of *NtMYB12*.

## Discussion

### *NtMYB12* is Involved in Flavonol Biosynthesis in Tobacco

In this study, we report a new PFG-type MYB transcription factor, *NtMYB12*, as a gene that regulates flavonol production in tobacco; this is similar to reports for *PtMYB12* and *AtMYB12* (**Figures 1** and **2**). There are various kinds of flavonol glycosides in plants. When *NtMYB12* was expressed at high levels in tobacco, significant increases were observed in two flavonols, rutin and kaempferol glycoside (**Figures 7A**, **B**). This result was consistent with those reported by Luo et al. (2008) and Zhai et al. (2019)

Previously, we reported that flavonols accumulate in tobacco plants grown under Pi deficiency (Jia et al., 2015). In the present study, the flavonol concentration of *NtMYB12* transgenic tobacco significantly increased under HP and LP conditions. Remarkably, we also found that the anthocyanin concentration and the relative transcript levels of anthocyanin biosynthesis genes *NtDFR* of *35S:NtMYB12* plants did not significantly increase under either LP or HP conditions (**Figure S3**), suggesting that *NtMYB12* plays a crucial role in the flavonol synthesis pathway. The roles of *CHS* and *FLS* in flavonol biosynthesis have been reported in other plants such as *Arabidopsis*, pear, and tomato. In this study, we also found that the expression patterns of the genes encoding *CHS*, *CHI*, and *FLS* were consistent with the patterns of flavonol accumulation in tobacco leaves (**Figures 7C**–**F**). Moreover, *NtCHS*-overexpressing plants had significantly increased flavonol concentration and a similar phenotype to *NtMYB12-*overexpressing plants (**Figures 8** and **S2**) under low Pi stress conditions, suggesting that *NtCHS* is a crucial downstream target gene of *NtMYB12*.

### Overexpression of *NtMYB12* Enhances Plant Resistance to Low Pi as Part of a Complex Network

Previous studies showed that *MYB* TFs are involved in various biotic and abiotic stresses (Abe, 2002; Agarwal et al., 2006; Yang et al., 2018; Zhao, 2019). Dai et al. (2012) first reported that *OsMYB2P-1*, an R2R3-MYB transcription factor, is involved in the regulation of phosphate-starvation responses in rice. Overexpression of *OsMYB2P-1* led to greater expression of high-affinity phosphate transporter genes (*Pht1*) such as *OsPT6*, *OsPT*8, and *OsPT10* under Pi-sufficient conditions, implying that *OsMYB2P-1* is a key transcriptional factor associated with Pi-starvation signaling in rice. Until now, the function of the MYB members involved in the Pi-starvation signaling pathway was still unclear in tobacco. In our previous study, a large number of flavonoid biosynthesis-related genes were cloned in tobacco (data not shown). However, we found that only the expression of *NtMYB12* was abundantly expressed under Pi deficiency. In this study, we further investigated the function of *NtMYB12* in the Pi signaling pathway and found that overexpression of *NtMYB12* significantly increased Pi and total P concentration, suggesting that this gene participates in the Pi signaling pathway in tobacco (**Figures 2**, **3**, and **6**).

Some studies showed that overexpression of the rice phosphate transporter gene *OsPT2* enhanced soybean tolerance to low phosphorus levels (Chen et al., 2015). Similarly, in a previous study, we observed that overexpression of *OsPT8* enhanced tobacco tolerance to phosphorus starvation, which may be a consequence of the increased expression of *NtPT1* and *NtPT2* and higher Pi uptake by tobacco roots (Song et al., 2017). In this study, overexpression of *NtMYB12* increased *NtPT1* and *NtPT2* expression in tobacco plants growing under Pi-sufficient conditions, suggesting that *NtMYB12* may be involved in the downstream regulation of tobacco Pi transporters under Pi restrictions (**Figure 6**). In our previous studies, we found that overexpression of *NtPT2* could enhance resistance to low Pi stress (Jia et al., 2017; Jia et al.,2018; Luo et al., 2019). Remarkably, in this work, we found that *35S:NtPT2* transgenic tobacco increased not only the Pi concentrations but also the flavonol concentration under low Pi stress conditions, showing a similar phenotype to *NtMYB12-*overexpressing plants at low Pi stress (**Figures 4**, **8**, and **S2**), suggesting that *NtMYB12* enhances resistance to low Pi stress by regulating the phosphate transporter *NtPT2* in tobacco plants. In addition, we also found that the expression level of flavonol synthesis-related genes (*NtCHS* and *NtFLS*) significantly increased in *35S:NtPT2* transgenic tobacco under both HP and LP conditions (**Figure S4**), implying that the high-affinity phosphate transporter *NtPT2* might be involved in regulating the flavonol biosynthetic pathway; this needs further study.

Previous studies have shown that flavonoids are powerful antioxidants and are involved in abiotic stress responses (Martinez et al., 2016; Chen et al., 2019). We found that ROS accumulation was decreased in *NtMYB12* transgenic tobacco under low Pi treatment (**Figure 5**), which was consistent with the concentration of MDA. Thus, it is likely that *NtMYB12* may promote low Pi tolerance at least partially through the antioxidant activity of flavonoids.

**Figure 9:**
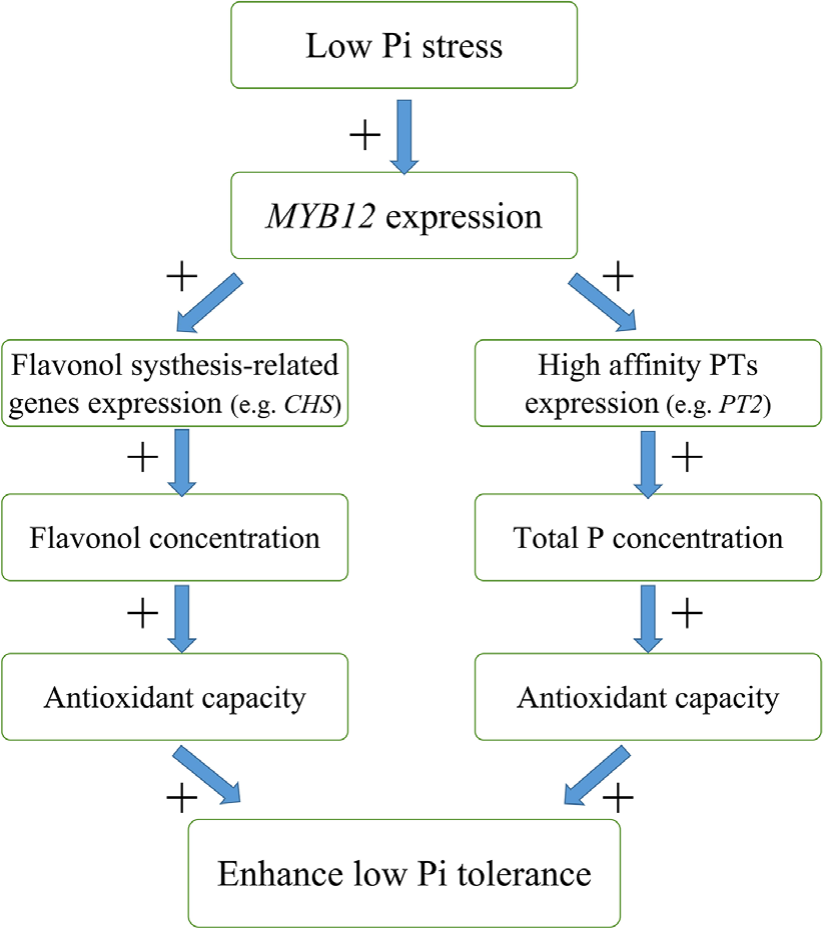
Possible model for the mechanism of interaction between *NtMYB12*, flavonol synthesis, and total P concentration under low Pi stress conditions in tobacco. The model is based on the results presented here. + indicates positive regulation.

In this study, we found that overexpression of *NtMYB12* increases flavonol concentration, enhances the activity of enzymatic and non-enzymatic antioxidant systems, and increases the total P concentration, thus improving the ability of plants to cope with low Pi stress. Based on our results, we propose a possible model for the regulation mechanism of *NtMYB12* in the Pi signaling pathway in tobacco under low Pi conditions (**Figure 9**): (1) *NtMYB12* expression in tobacco is up-regulated, (2) then, the expressions of flavonol synthesis-related genes and *Pht1* family genes are significantly up-regulated (3) increasing flavonol and total P concentration; (4) as a result, the antioxidant capacity rises; these events may result in enhanced tolerance to low phosphate levels. As a whole, our results suggest that *NtMYB12* might function in a complex network that leads to enhanced tolerance to Pi starvation.

## Conclusion

We characterized the functions of *NtMYB12* as an enhancement factor in low Pi responsiveness. The expression of *NtMYB12* was up-regulated by low Pi stress, resulting in the activation of *NtCHS* and *NtPT2* transcription, followed by the accumulation of flavonols and total P and then an enhancement of tolerance to low Pi stress. A better understanding of the regulatory networks underlying flavonol biosynthesis and plant responses to Pi deficiency could help in the breeding of plant varieties with improved phosphorus use efficiency.

## Data Availability Statement

All datasets generated for this study are included in the article/**Supplementary Material**.

## Author Contributions

HJ, DW, and CY designed this study. ZS and YL did the main experimental work. All of the authors carried out the field experiments. WW and YL made a modification of the manuscript. HJ and ZS wrote the manuscript. All of the authors read and approved this manuscript.

## Funding

This work was supported by the National Natural Science Foundation of China (31301837), the Outstanding Young Teachers Development Program of Henan Province (2019GGJS043), and the Foundation of the Zhoukou Branch of Henan Tobacco Company (2019411600240174).

## Conflict of Interest

The authors declare that the research was conducted in the absence of any commercial or financial relationships that could be construed as a potential conflict of interest.
