# Supplementary material for: NtMYB12 Positively Regulates Flavonol Biosynthesis and Enhances Tolerance to Low Pi Stress in Nicotiana tabacum
Source: Front Plant Sci. 2020 Jan 24;10:1683. doi: 10.3389/fpls.2019.01683 (PMC6993060; doi:10.3389/fpls.2019.01683)
Supplement: Supplementary file 1 [file Table_1.docx]

SUPPLEMENTARY MATERIAL

**Table S1.** Primers used to amplify the full-length of *NtMYB12* and relative genes for RT-qPCR**.**

| **Gene** | **No. ID** | **Primer sequences F (5’ to 3’)** | **Primer sequences R (5’ to 3’)** |
| --- | --- | --- | --- |
| *35S:NtMYB12* | XM_016624824 | AACCGGTACCATGGGAAGAGCACCTTGTTGT | TTAACCTAGGCTAAGACAAAAGCCAAGCGACAA |
| *NtMYB12*  *-YXB* | XM_016624824 | CAGTCGTCTCACAACATGGGAAGAGCACCTTGTTG | CAGTCGTCTCATACAAGACAAAAGCCAAGCGACAAG |
| *35S:NtPT2* | AB042951 | CGAGCTCATGTCTGCAGATAACAATC | GCTCGAGTCATTCTTCAGTTATAGTTTCC |
| *35S:CHS* | AF311783 | ACGAGCTCATGGTGACCGTCGAGGAATTTCG | CAGGTACCAAGTAGCAACACTGTGGAGAAC |
| *NtMYB12* | XM_016624824 | AGCTCATGGTTAGAAGGTAAC | CATTCCATCCCACTATCTGAAC |
| *NtCHS* | AF311783 | CGTAGGGCGCAATGTGCCGAG | CGAAAATAATAATCAGGATAAG |
| *NtCHI* | XM_016600175 | GAATTACGTGTTCCCATCAAC | ACAAACTTCCCTTCAATTTC |
| *NtF3H* | AF036093 | GTGGACACGAAGATGTTATCAG | TGAAGATGGCTAGAAACGATG |
| *NtFLS* | DQ435530 | CTCAGAGTGCAACAACCCTAAC | CTGGCTGCTCGTTCTCTGAC |
| *NtPT1* | AB020061 | ATGGGCTTCTTTACTGATGC | TTCCCATTTTATCTCCGAGCC |
| *NtPT2* | AB042951 | CTTGGGCGTTTATACTACACC | CCATGATAATCAAAGTCATACC |
| *NtDFR* | [AB289448](http://www.ncbi.nlm.nih.gov/nucleotide/164454778?report=genbank&log$=nucltop&blast_rank=1&RID=MK102TC0016) | GTTCACGCTACTGTTCGTGATCC | CAAGTCCGCTTTCCACAGCG |
| *NtL25* | L18908 | CCGTCCAAAAAATCTGACCC | TCTTCAAAGTCTTAGGTCGG |

**Figure S1**


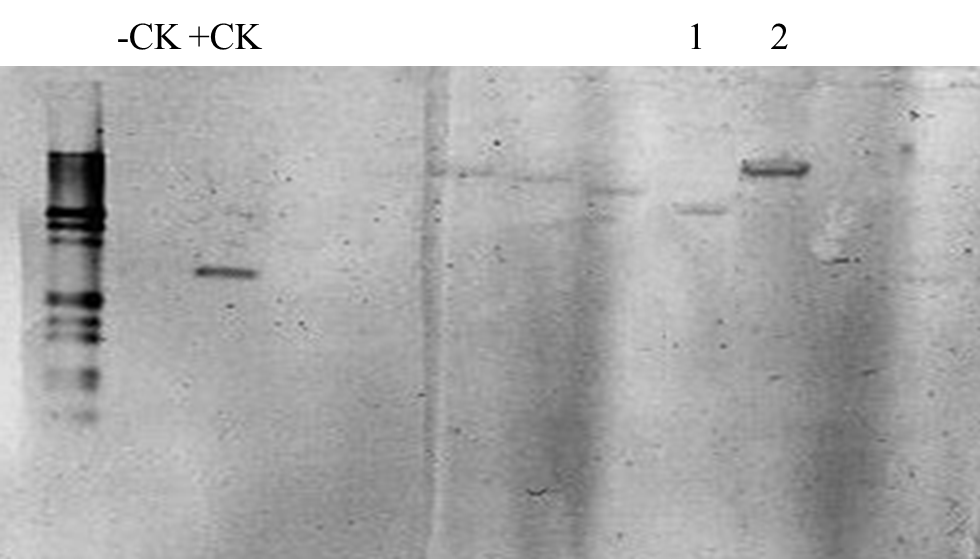


**Figure S1 Southern blot analysis of *35S:NtMYB12* plant and WT.**

Hygromycin gene was used as probe and ﬁve micrograms of genomic DNA of each sample were digested by SacI, and separated by agarose gel. -CK, negative control; +CK, positive control. 1, *35S:NtMYB12-1*; 2, *35S:NtMYB12-2.*

**Figure S2**


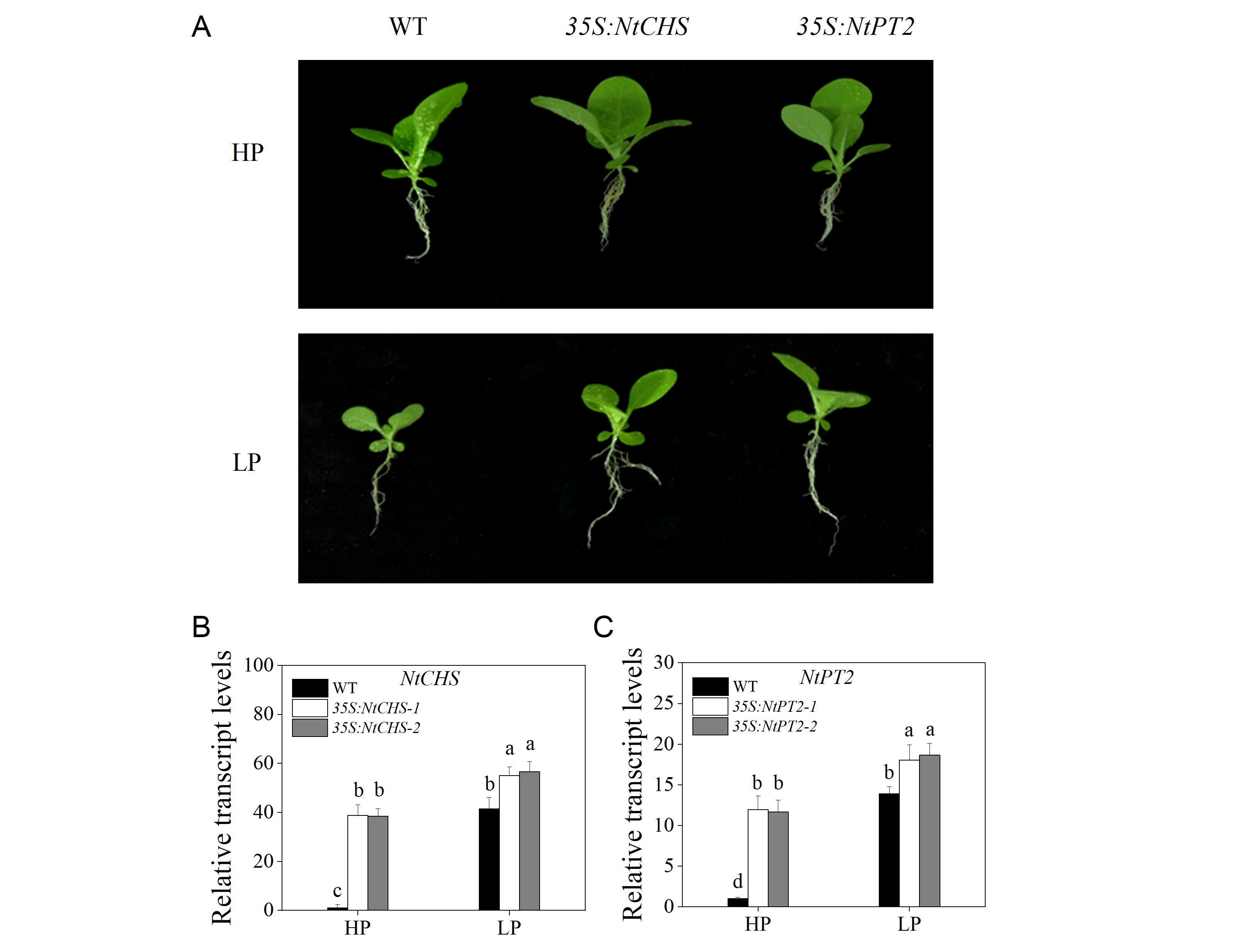


**Figure S2 Expression of *NtCHS and NtPT2* in transgenic tobacco and characterization of the WT and transgenic tobacco plants.**

**(A),** Characterization of the transgenic tobacco *35S:NtCHS* and *35S:NtPT2* under HP and LP conditions. **(B-C)**, Relative transcript levels of *NtCHS and NtPT2* in transgenic plants. Sixteen-day-old seedlings were transferred to the full-strength culture solution supplied with high Pi (HP; 1mM Pi) or low Pi (LP; 0.02mM Pi) for 14 days. Data are the means ± SDs of three biological replicates. Different letters indicate significant differences (*P* < 0.05).

**Figure S3**


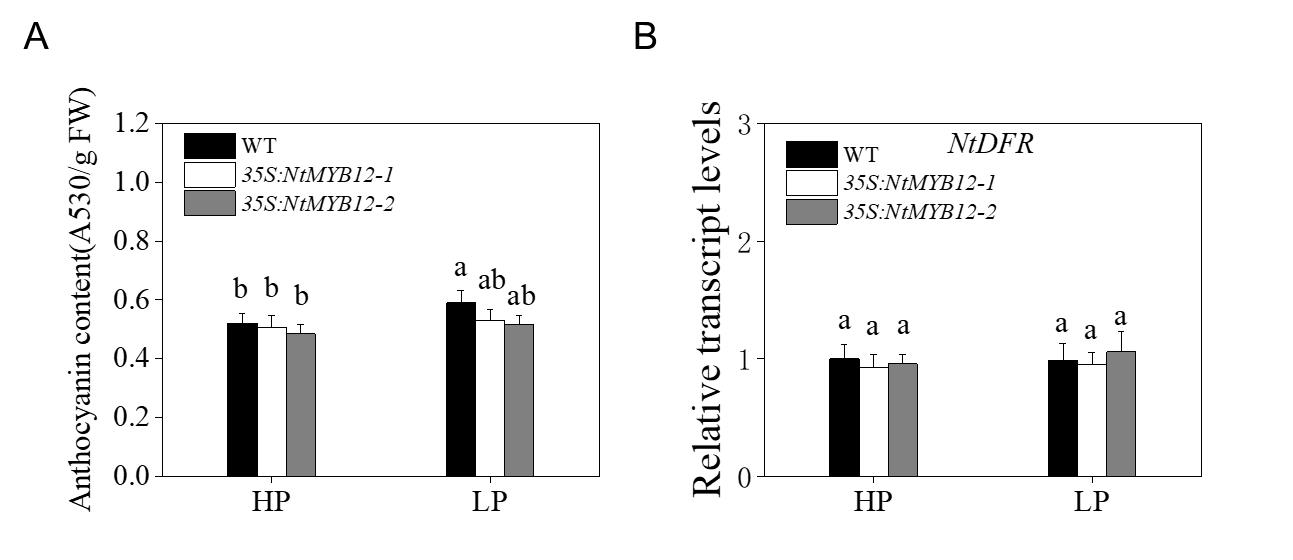


**Figure** **S3** **Anthocyanin concentration and relative transcript levels of anthocyanin biosynthesis genes *NtDFR* in WT, *35S:NtMYB12* plants.**

**(A)**, Anthocyanin concentration of WT and transgenic plants in tobacco. **(B)**, the relative transcript levels of anthocyanin biosynthesis genes *NtDFR*. Sixteen-day-old seedlings were transferred to the full-strength culture solution supplied with high Pi (HP; 1mM Pi) or low Pi (LP; 0.02mM Pi) for 21 days. FW, fresh weight. *DFR:* dihydroflavonol reductase. Data are the means ± SDs of five biological replicates. Different letters indicate significant differences (*P* < 0.05).

**Figure S4**


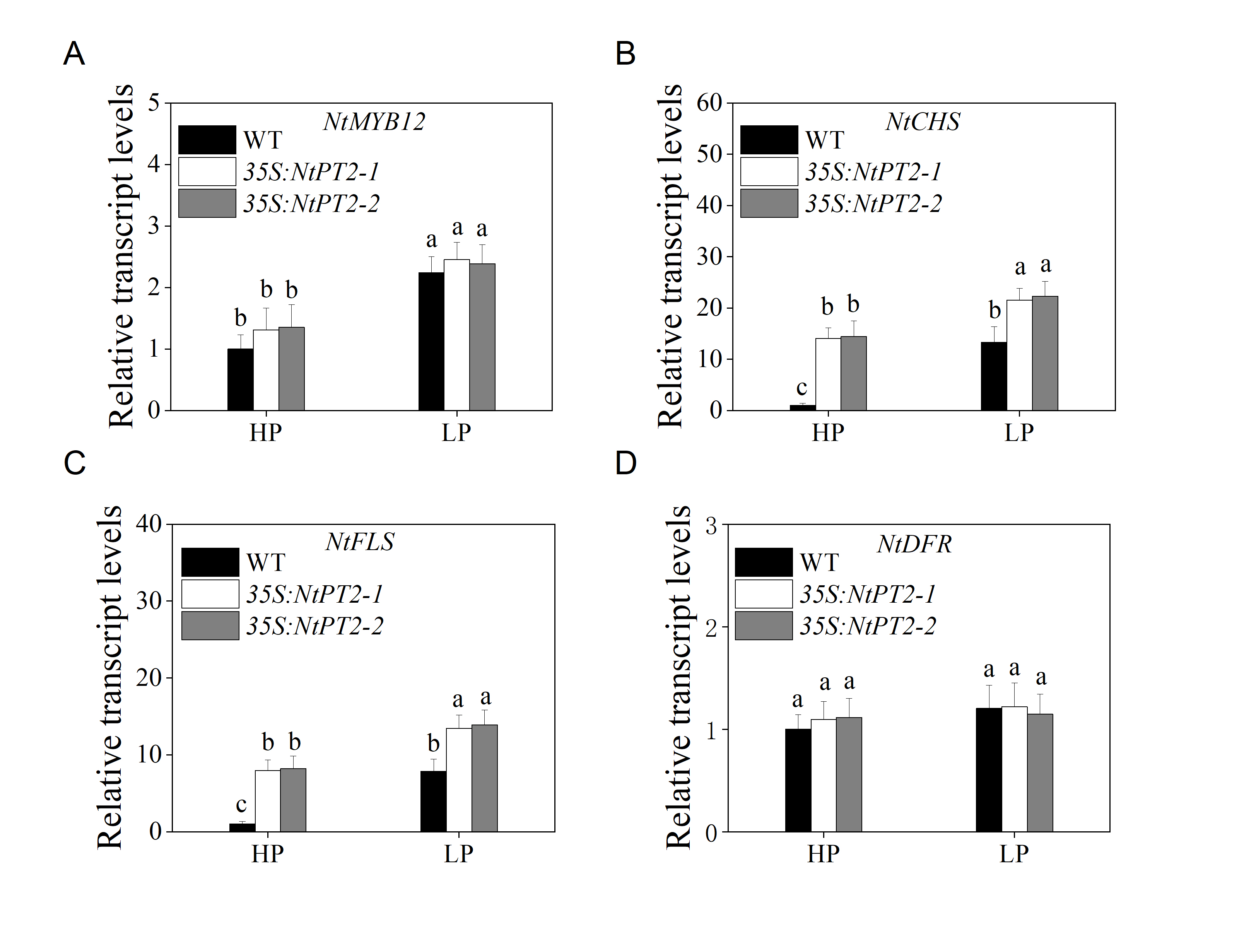


**Figure S4 Relative transcript levels of *NtMYB12*, *NtCHS*, *NtFLS* and *NtDFR* in *35S:NtPT2* plants.**

**(A-D)**, Relative transcript levels of *NtMYB12*, *NtCHS*, *NtFLS* and *NtDFR* in *35S:NtPT2* plants. Sixteen-day-old seedlings were transferred to the full-strength culture solution supplied with high Pi (HP; 1mM Pi) or low Pi (LP; 0.02mM Pi) for 14 days. Data are the means ± SDs of three biological replicates. Different letters indicate significant differences (*P* < 0.05).
